# Supplementary material for: STAT1 as a tool for non-invasive monitoring of NK cell activation in cancer
Source: Commun Biol. 2024 Sep 30;7:1222. doi: 10.1038/s42003-024-06917-9 (PMC11442705; doi:10.1038/s42003-024-06917-9)
Supplement: Supplementary file 2 — Supplementary information [file 42003_2024_6917_MOESM2_ESM.pdf]

Supplementary Information

STAT1 as a tool for non-invasive monitoring of NK cell activation in cancer

Jin Young Min [a,1], Hye Min Kim [a,b,1], Hyunseung Lee [a], Mi Young Cho [a], Hye Sun Park [a], Sang-Yeop Lee [a,b], Min Sung Park [a,b], Sang Keun Ha [b,c], Donghwan Kim [c], Hye Gwang Jeong [d], Tae-Don Kim [b,e], Kwan Soo Hong [a,f,*] and Eun Hee Han [a,b,*]

[a] Biopharmaceutical Research Center, Ochang Institute of Biological and Environmental Science, Korea Basic Science Institute (KBSI), Cheongju 28119, Republic of Korea

[b] Korea University of Science and Technology (UST), Daejeon, 34113, Republic of Korea

[c] Food Functionality Research Division, Korea Food Research Institute, Jeollabuk-do, 55365, Republic of Korea

[d] College of Pharmacy, Chungnam National University, Daejeon 34134, Republic of Korea.

[e] Korea Research Institute of Bioscience and Biotechnology, Daejeon 34141, Republic of Korea

[f] Department of Chemistry, Chung-Ang University, Seoul 06974, Republic of Korea.

[1] These authors contributed equally to this work.

* These authors jointly supervised this work.

Correspondence: E-mail: kshong@kbsi.re.kr; heh4285@kbsi.re.kr

Quantitative PCR

RNAs were isolated from human NK cells infected with pGF-GAS or pGF-empty lentiviral

particles using a RNeasy Mini Kit (Qiagen, Venlo, the Netherlands). cDNAs were then synthesized using a High-Capacity cDNA Reverse Transcription Kit (Applied Biosystems, Carlsbad, CA, USA). For real-time PCR, SYBR Green PCR Master Mix (Applied Biosystems) was used. Primers for human GAPDH (Forward; 5’- CGGGAAACTGTGGCGTGATG-3`, reverse; 5`-ATGACCTTGCCCACAGCCTT-3’) and GAS (Forward; 5’- ACGCCCGACTAC AACTAAAGAATTACA-3`, reverse; 5`- CCTCCCACCGTACACGCCTA-3’) and IFNγ (Forward; 5’- ATGAAATATACAAGTTATATCTTGGCTTT-3`, reverse; 5’- GATGCTCTT CGACCTCGAAACAGCAT-3’) were used. Samples were run as duplicates in MicroAmp Optical 96-well reaction plates (Applied Biosystems) with a 7900HT Fast Real-Time PCR System (Applied Biosystems). Data were analyzed using SDSv2.3 software (Applied Biosystems). Accumulated PCR products were directly detected by monitoring increases in SYBR reporter dye fluorescence and DNA electrophoresis.

Chromatin immunoprecipitation (ChIP) assay

Chromatin immunoprecipitation was performed using a ChIP kit (Millipore, 17-295) following the manufacturer’s instructions. This assay evaluated STAT1 binding to the GAS promoter response element in GAS-NK and empty-NK cells. For this, cells (with or without IFNα treatment) were lysed in digestion buffer containing 50 mM Tris-Cl (pH 7.6), 1 mM $CaCl_2$, 0.2% Triton X-100, 5 mM butyrate, 1× protease inhibitor cocktail, and 0.5 mM PMSF. The lysate was then treated with 0.3 U of micrococcal nuclease (MNase; Sigma-Aldrich, St. Louis, MO, USA) and incubated at 37°C for 5 minutes. The reaction was stopped using 50 mM EDTA and RIPA buffer and incubated for 16 hours. Approximately 3 μg of primary antibody against STAT1 (#14994S, Cell Signaling Technology) was then coupled to Dnabeads Protein A beads (#9733, Invitrogen) and incubated with the solution at 4°C for 16 hours. Normal rabbit IgG (sc-2025, Santa Cruz Biotechnology) served as the control. Finally, DNA was extracted,

processed, and used for real-time PCR analysis as per the ChIP assay kit instructions.

NK cytotoxicity assay

To measure NK cell cytotoxicity, we employed the calcein AM release assay. Target cancer cells (K562) were first labeled with calcein green (Thermo Fisher Scientific) for 30 minutes at 37°C. Subsequently, these labeled cancer cells (1×10^4 cells) were co-cultured with effector cells (empty and GAS-NK cells) at an Effector:Target (E:T) ratio of 2:1 in 96-well round-bottom plates for a duration of 4 hours. Calcein release into the supernatant was quantitatively measured using a multimode microplate reader (Spectra Max M5, Molecular Device Co., San Jose, CA, USA). To determine the maximal release, 1% Triton X-100 (Sigma, St. Louis, MO, USA) was added to the target cells. For assessing spontaneous release, the culture medium alone was added to the target cells. The specific lysis rate was calculated using the following formula: [(Sample Release - Spontaneous Release) / (Maximum Release - Spontaneous Release)] × 100%.

Lactate Dehydrogenase (LDH) assay

To investigate the effect of NK cells on cancer cell viability, 5000 A549 cells per well were incubated with empty-NK or GAS-NK cells at effector-to-target (E:T) ratios of 5:1 and 10:1 for 4 hours. After 4 hours, 50 μl of medium from each well was transferred into a new 96-well plate. The content of LDH in the medium was measured using the Cytotoxicity Detection Kit PLUS (LDH) (4744926001, Sigma) according to the manufacturer's instructions.

Ingenuity pathway analysis

Ingenuity Pathway Analysis (IPA) (Ingenuity Systems, www.ingenuity.com, Redwood City, CA, USA) is an online software package used to identify canonical pathways, gene networks,

and categorize specific physiological processes. The Ingenuity Pathway Knowledge Base was utilized for a deeper analysis of the global molecular network and the discovery of interactions among differentially expressed genes.

Laser microdissection and real-time PCR analysis for tumor-adjacent cell classification and GAS-NK cell homing

To distinguish between normal and cancerous cells adjacent to tumors in lung cancer metastatic tissue, we utilized laser capture microdissection (LCM). Using the LMD 6 system from Leica, approximately six areas of both normal and cancerous cells in lung metastasis tissue administered GAS-NK cells were precisely isolated. The dissected tissues were collected into PCR tubes and stored at -80°C for subsequent RNA isolation. For the extraction of small quantities of RNA, the PicoPure RNA Isolation Kit (Applied Biosystems, Cat# KIT2024) was employed, followed by cDNA synthesis using the SuperScript IV Single Cell/Low Input cDNA PreAmp Kit (Invitrogen, Cat# 11752048). This method enables us to perform a detailed analysis of the tumor microenvironment and the homing of GAS-NK cells at the tumor site. By employing real time-PCR with primers specific for GAPDH, E-cadherin, GAS, CD56, NKG2D, TNFα, and IL-6 we aim to gain insights into the gene expression patterns in the metastatic lung tissue, further elucidating the interaction between the tumor, its microenvironment, and the infiltrating GAS-NK cells. The primers used were specific for human GAPDH (forward: 5'-CGGGAACTGTGGCGTGATG-3', reverse: 5'-ATGACCTTGCCCACAGCCTT-3'), human E-cadherin (forward: 5'-TGGAGGAATTCTTGCTTTGC-3', reverse: 5'-CGCTCTCCTCCGAAGAAAC-3'), CD56 (forward: 5'-CATCACCTGGAGGACTTCTACC-3', reverse: 5'-CAGTGTACTGGATGCTCTTCAGG-3'), NKG2D (forward: 5'-ACCCAACCTACTAACAATAA-3', reverse: 5'-TACCGCTGGTGTAATCTC-3'), TNFα

(forward: 5'- AGCACTGAAAGCATGATCCG -3', reverse: 5'- CCGATCACTCCAAAGTGCAG -3') and IL-6 (forward: 5'- AGACAGCCACTCACCTCTTC -3', reverse: 5'- AGTGCCTCTTTGCTGCTTTC -3'). PCR reactions were carried out using the 7900HT fast real-time PCR System and the resulting data were then analyzed with the SDS v2.3 software by Applied Biosystems.

Maintenance of human induced pluripotent stem cells (hiPSCs)

Human hiPSCs (Cellartis Human hiPSC Cell Line 18, ChiPSC18; cat. Y00305; Takara Bio Europe AB, Goteborg, Sweden) were meticulously maintained in a feeder-free environment following the manufacturer's guidelines. Cells were cultured on surfaces coated with COAT-1 (cat. Y30012, Takara Bio Europe AB), ensuring optimal adhesion and growth. The culture conditions included a humidified atmosphere with 5% CO2 at 37°C, with daily medium changes to fresh Cellartis DEF-CS 500 complete medium. This medium comprised Cellartis DEF-CS 500 Basal Medium (cat. Y30011), enhanced with DEF-CS GF1 (1:333 dilution; cat. Y30016) and DEF-CS GF2 (1:1000 dilution; cat. Y30016). Additionally, DEF-CS GF3 (also diluted 1:1000; cat. Y30016) was specifically incorporated into the complete medium during critical phases such as cell thawing, passaging, or cryopreservation, but it was omitted during regular medium refreshment to maintain optimal cell health and promote steady growth.

hiPSC-derived normal tissue organoid formation

For generating human liver organoids, iPSCs were detached using accutase and seeded onto laminin-coated 6-well plates. The culture underwent staged medium changes, starting with RPMI 1640 containing activin A and BMP4. Fetal calf serum was added in subsequent days for differentiation. From days 4 to 6, cells were cultured in Advanced DMEM/F12, supplemented with B27, N2, FGF4, and CHIR99021, for hepatic specification. The medium

was refreshed daily, and cells were maintained at 37°C in a 5% CO_2 atmosphere. After detachment, foregut cells were preserved. For organoid formation, cells were embedded in Matrigel and cultured in organoid formation medium with specific growth factors. Following a 4-day culture, hepatic organoids were harvested from Matrigel and transferred to Ultra-Low Attachment Multiwell Plates in liver maturation media for 10 days, with medium refreshed every two days. Human intestinal organoids were derived from iPSCs using the STEMdiff™ Intestinal Organoid Kit, provided by STEMCELL Technologies (a biotechnology company based in Vancouver, Canada). These organoids were maintained in STEMdiff™ Intestinal Organoid Growth Medium, with media changes every 2-3 days, ensuring optimal growth and development at 37°C and 5% CO_2.

Supplementary Figure Legends

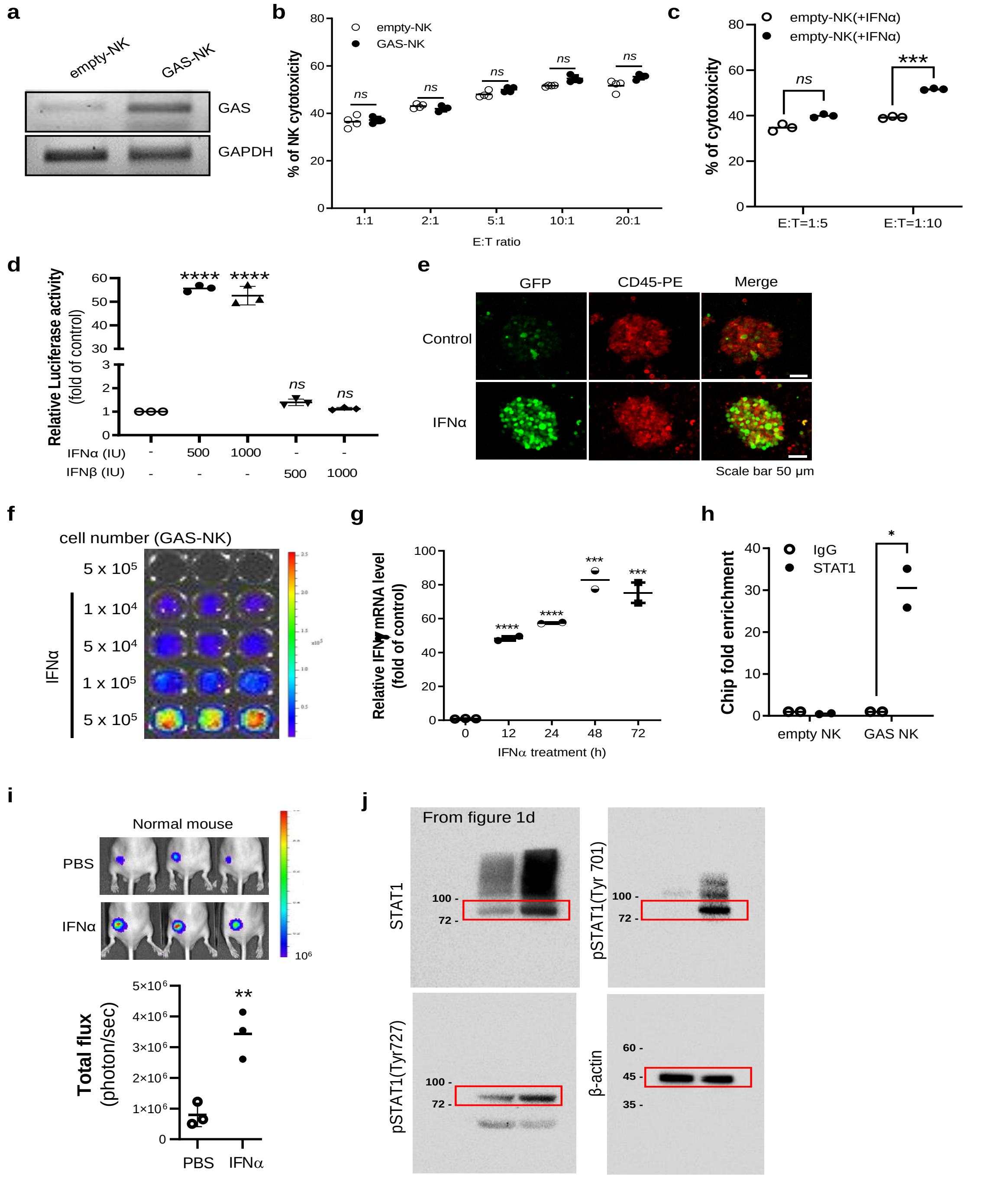

Supplementary Fig. 1. Comprehensive validation of GAS-NK cell line production. (a) Quantitative PCR analysis of GAS element expression in NK cells, contrasting empty-NK and GAS-NK cells. (b) Measurement of K562 cancer cell cytotoxicity by empty or GAS-NK cells was conducted using a calcein AM-based cytotoxicity assay at various effector-to-target (E:T) ratios for 4 hours. ns, not significant. (c) Analysis of NK cell cytotoxicity by IFNα: GAS-NK

and empty-NK cells were pretreated with 500 IU of IFNα for 16 hours, then co-cultured with A549 cells for 4 hours. The cancer cell killing ability of NK cells was measured by LDH assay (E:T ratio = 5:1, 10:1). n = 3 (for all samples). (d) Assessment of changes in luciferase activity in GAS-NK cells post-IFNα and IFNβ treatment. (e) Fluorescence imaging demonstrating GFP expression in CD45-PE-stained GAS-NK cells, post-24-hour IFNα treatment. (f) Bioluminescence imaging corresponding to the quantity of GAS-NK cells post-IFNα treatment. (g) Gene expression kinetics of IFNγ quantified by qPCR with 5 timepoints in GAS-NK cells. n = 3 (samples for all time points). (h) Chromatin immunoprecipitation (ChIP) analysis targeting the GAS promoter motif bound to STAT1, with normal IgG as a negative control (statistical significance: *$P < 0.05$). GAS-NK cells were treated with IFNα for 24 hours before lysis. (i) In vivo evaluation of GAS-NK cell activation post-subcutaneous injection into mice, followed by abdominal IFNα injection and luminescence imaging. (j) Extended blot imagery from Fig. 1d, showcasing STAT1 protein expression and phosphorylation levels in GAS-NK cells post-IFNα treatment.

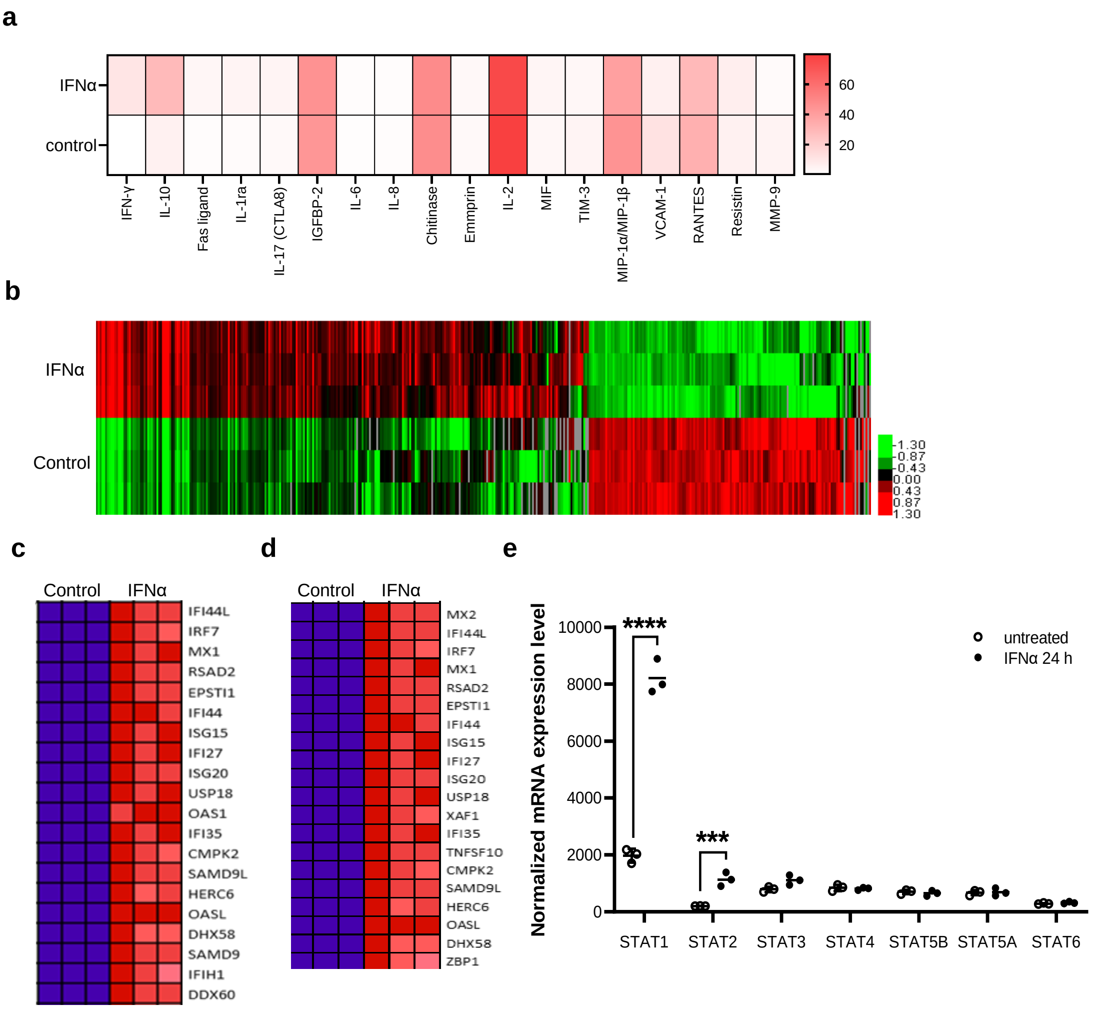

Supplementary Fig. 2. Detailed analysis of GAS-NK cell responses. (a) Tile plot representing the array of cytokines secreted by GAS-NK cells post-IFNα treatment, as shown in Fig. 2a. The intensity variation, from white to red, indicates the range of cytokine expression. (n = 2, control and IFNα). (b) Heatmap analysis of genes positively enriched in GAS-NK cells following 24-hour IFNα treatment compared to the control group, highlighting significant differences in gene expression. The heatmap is based on hierarchical clustering of the filtered gene data. (c, d) Gene lists for the Gene Set Enrichment Analysis (GSEA) shown in Figs. 2d and 2e, presenting the gene responses to IFNα (c) and IFNγ (d). (e) RNA expression levels of the STAT family in GAS-NK cells treated with IFNα compared to untreated cells, highlighting the activation of STAT1 signaling in IFNα-treated GAS-NK cells. (n = 3, all other samples).

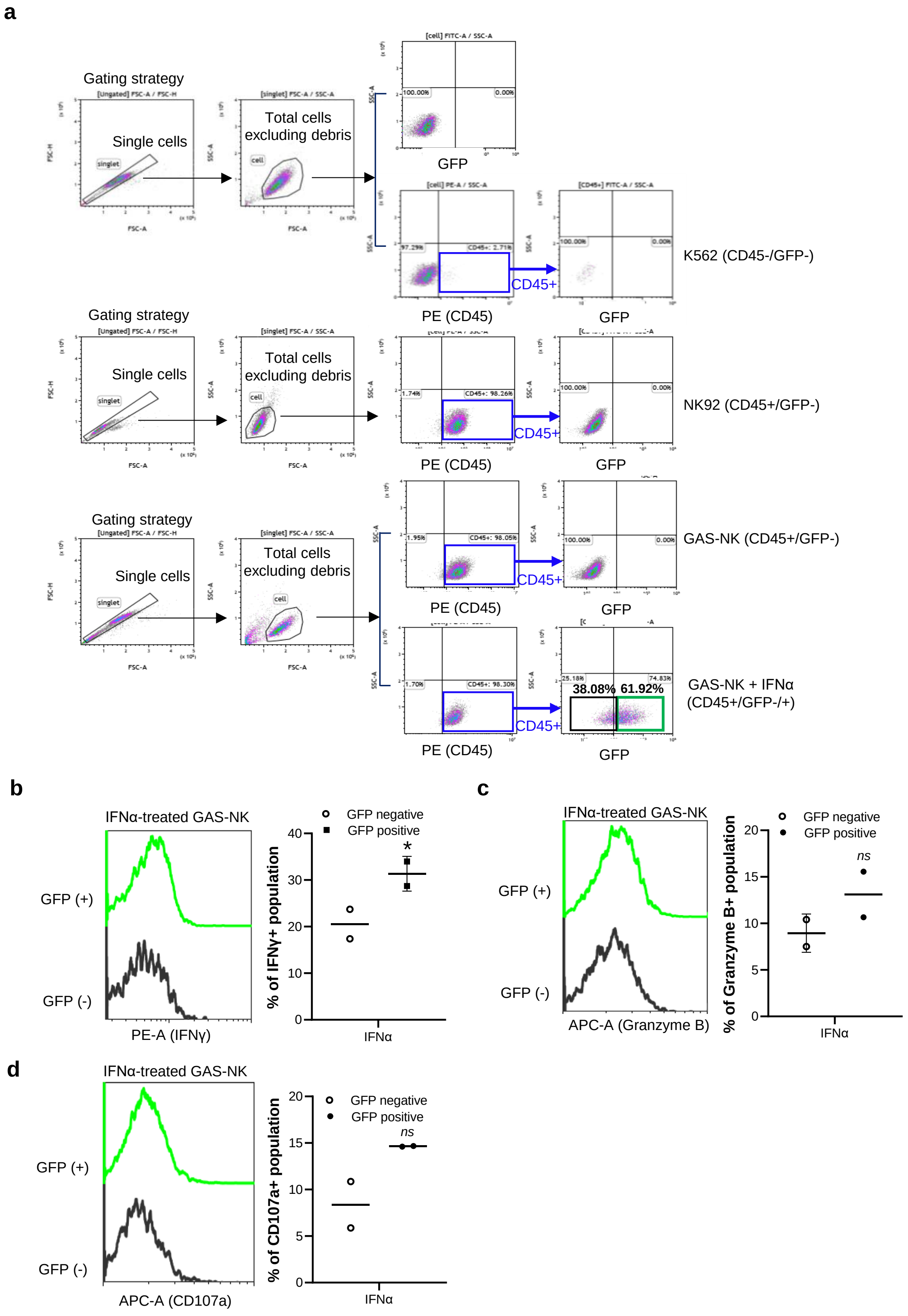

Supplementary Fig. 3. Evaluation of IFNα-treated GAS-NK cell populations. (a) Gating strategy used to distinguish GFP-positive and GFP-negative populations. Individual panels

show representative flow cytometry analysis using a gating strategy to analyze CD45-PE and GFP populations. The bottom figure displays the flow cytometry results of GAS-NK cells treated with IFNα, classified into GFP-positive and GFP-negative groups based on GFP intensity in response to IFNα. The subsequent panels (b, c, d) display the analysis of fluorescence intensity for IFNγ (BioLegend, Cat #. 502509), Granzyme B (BioLegend, Cat #. 372204), and CD107a (BioLegend, Cat #. 328620) antibodies, respectively, in both GFP-negative and GFP-positive GAS-NK cell populations. This comparative approach facilitates the understanding of the differential expression of these key markers between the two populations. (n = 2, all other samples).

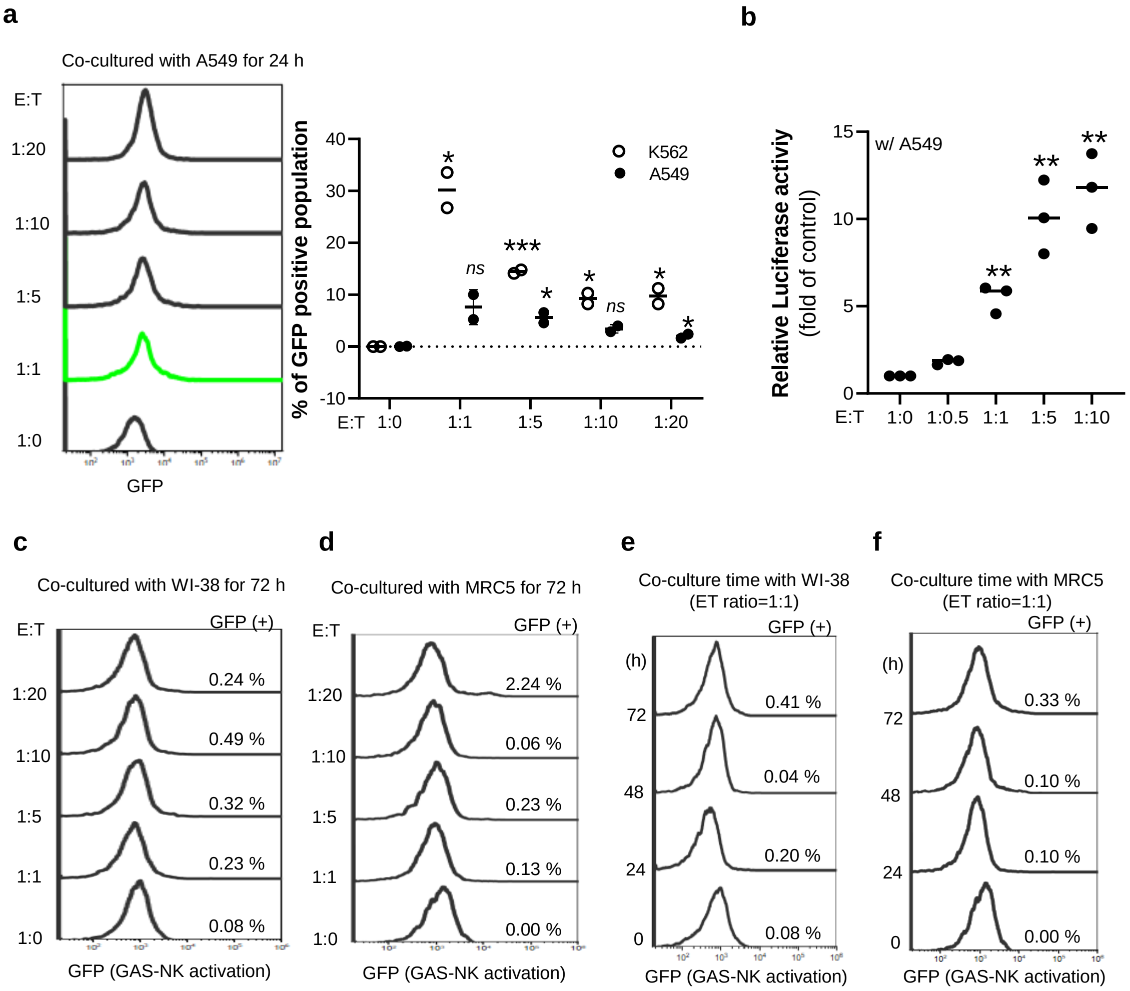

Supplementary Fig. 4. Detailed analysis of GAS-NK cell activation in co-culture settings. (a) Flow cytometry data displaying GFP expression levels in GAS-NK cells co-cultured with A549

cells at different E:T ratios. The quantitative graph compares these findings with those from the K562 co-culture in Fig. 3e. Statistical significance: $^*P < 0.05$; $^{***}P < 0.001$; ns, not significant. ($n = 2$, all other samples). (b) Quantitative luciferase activity in GAS-NK cells during A549 co-culture across E:T ratios (1:0, 1:0.5, 1:1, 1:5, 1:10). Statistical significance: $^{**}P < 0.01$ for all ratios. ($n = 3$, all other samples). (c-f) Confirmation of GAS-NK cell inactivation in co-culture with normal cell lines WI-38 and MRC-5, analyzed at various E:T ratios over 72 hours. Flow cytometry showed no significant change in GFP intensity, confirming the stability of GAS-NK cells in normal cell environments. ($n = 2$, all other samples).

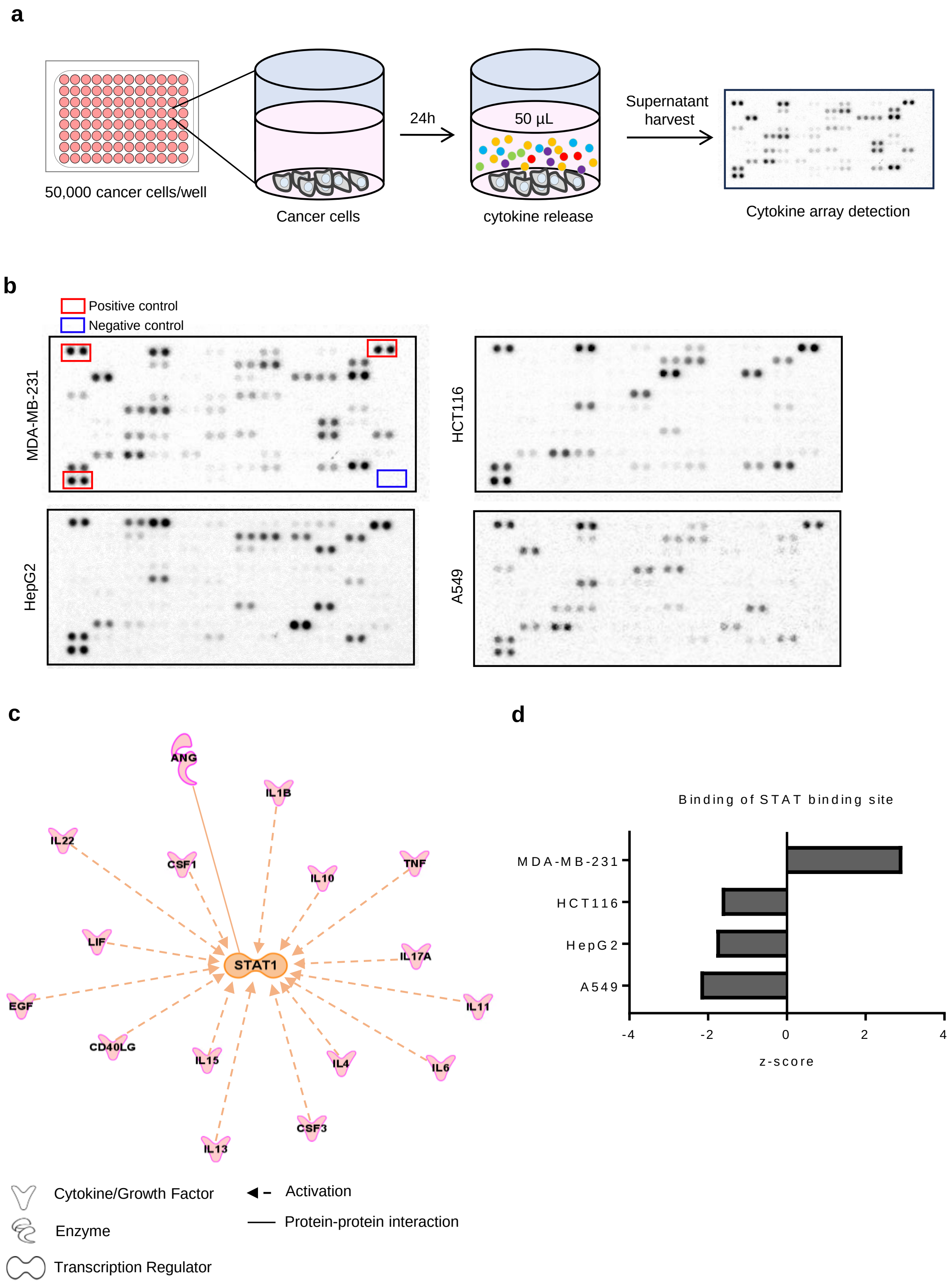

Supplementary Fig. 5. Cytokine profile analysis in supernatants from solid tumor cell lines and analysis of cancer cell cytokine profiles and network pathways. (a) Multiplex cytokine analysis was performed on various cancer cell lines, each comprising 50,000 cells in a 96-well

plate, to assess the cytokine secretion profile. (b) Blot images present the cytokine profiles in the supernatants of four different cancer cell lines: MDA-MB-231, HCT116, HepG2, and A549. The blot images highlight differences in cytokine levels, with red squares indicating the presence of specific cytokines, and blue squares marking the baseline or negative controls. This detailed cytokine analysis provides insights into the tumor microenvironment influenced by different cancer cell lines. (c) Analysis using the Ingenuity Pathway Analysis (IPA) tool on the cytokine profiles secreted by various solid tumors, specifically focusing on those related to STAT1 activation. The cytokine levels for each cancer cell line are represented, with the intensity of pink color indicating higher cytokine amounts. The analysis computes the density of cytokines relative to positive and negative controls. (d) IPA's disease and function pathway analysis based on increased cytokine production in each solid cancer cell line. The graphs display z-scores (x-axis) for the 'Binding of STAT bind site.'

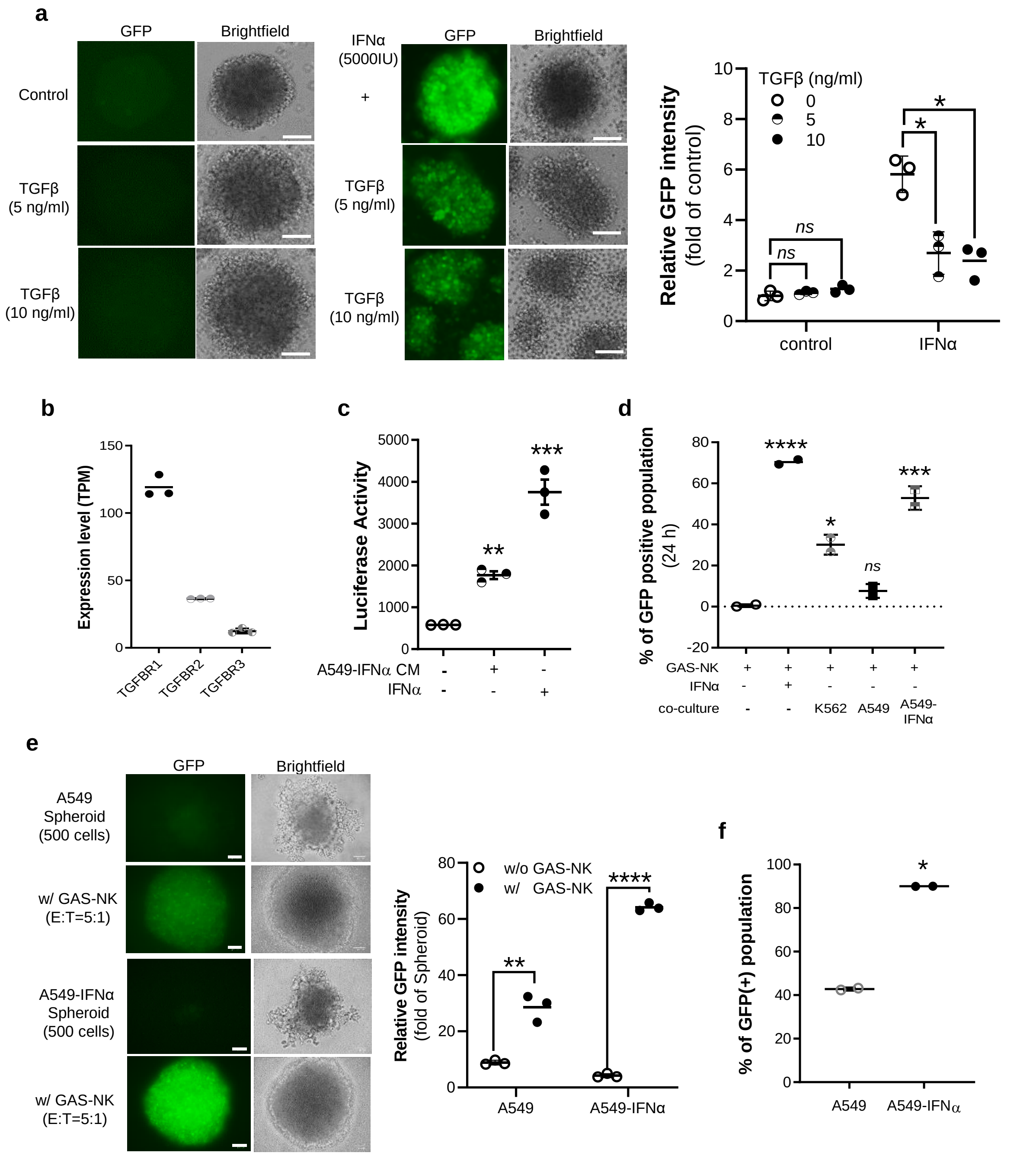

Supplementary Fig. 6. Assessing the impact of tumor microenvironment cytokines on GAS-NK cells. (a) GFP intensity changes in GAS-NK cells after treatment with TGFβ or IFNα, accompanied by a graph quantifying GFP intensity. Statistical significance is indicated (*P < 0.05; ns, not significant). (b) Mean gene expression levels (TPM) from RNAseq data for the TGFβ receptor family in GAS-NK cells. (n = 3, all other samples). (c) Luciferase activity in GAS-NK cells following 24-hour exposure to the A549-IFNα culture supernatant, with IFNα

serving as the positive control. Statistical significance is indicated (**$P < 0.01$; ***$P < 0.001$). (d) Percentage of GFP(+) population at 24 hours for GAS-NK co-cultured with IFNα treatment, K562, A549, and A549-IFNα cell lines. (n = 3, all other samples). (e) Images and a graph detailing GFP intensity in GAS-NK cells co-cultured with A549 or A549-IFNα spheroids for 24 hours at an E:T ratio of 1:1, highlighting significant differences (**$P < 0.01$; ****$P < 0.0001$). (n = 3, all other samples). (f) Graph quantifying the percentage of GFP-positive GAS-NK cells following co-culture with A549 or A549-IFNα for 24 hours at an E:T ratio of 1:1, with noted statistical significance (*$P < 0.05$). (n = 3, all other samples).

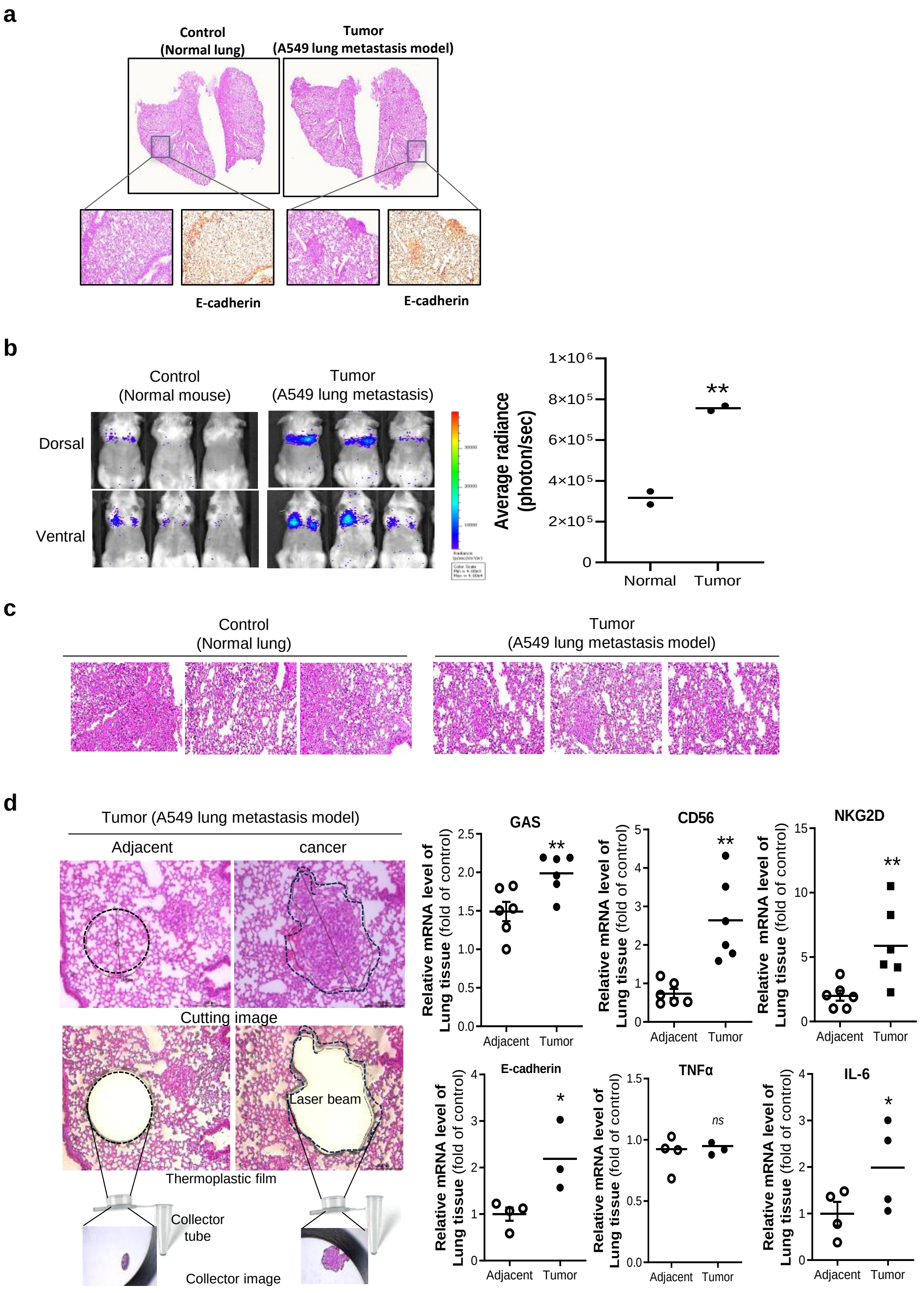

Supplementary Fig. 7. Evaluating GAS-NK cell activation in an A549 lung metastasis mouse model. (a) Representative combined H&E-stained image of a mouse lung with A549 metastasis, highlighting tumor enlargement, and an E-cadherin immunohistochemistry (IHC) image indicating tumor presence. (b) Process and results of injecting GAS-NK cells into the tail vein

of control and A549 metastasis model SCID mice, followed by luminescence imaging at the tumor site to quantify the activation of GAS-NK cells. Statistical significance is denoted (**$P < 0.01$). ($n = 3$, all other samples). (c) H&E staining images comparing normal lung tissue and lung tumor tissue in mice 24 hours after GAS-NK cell injection, illustrating the histopathological changes due to tumor metastasis. (d) Tissue images showing laser cutting, including adjacent (left side) and cancer cells (right side) of lung metastasis tissue administered GAS-NK cells. Schematic illustration of laser capture microdissection for sorting tissue. The laser micro-dissected cells were dropped by gravity into a 0.2 ml PCR tube cap. Real-time PCR analysis of GAS, CD56, NKG2D, E-cadherin, TNFα, and IL-6 for elucidating the interaction between the tumor and the infiltrating GAS-NK cells. Statistical significance is marked for observed differences in activation (*$P < 0.05$; **$P < 0.01$). ($n = 3$, all other samples).

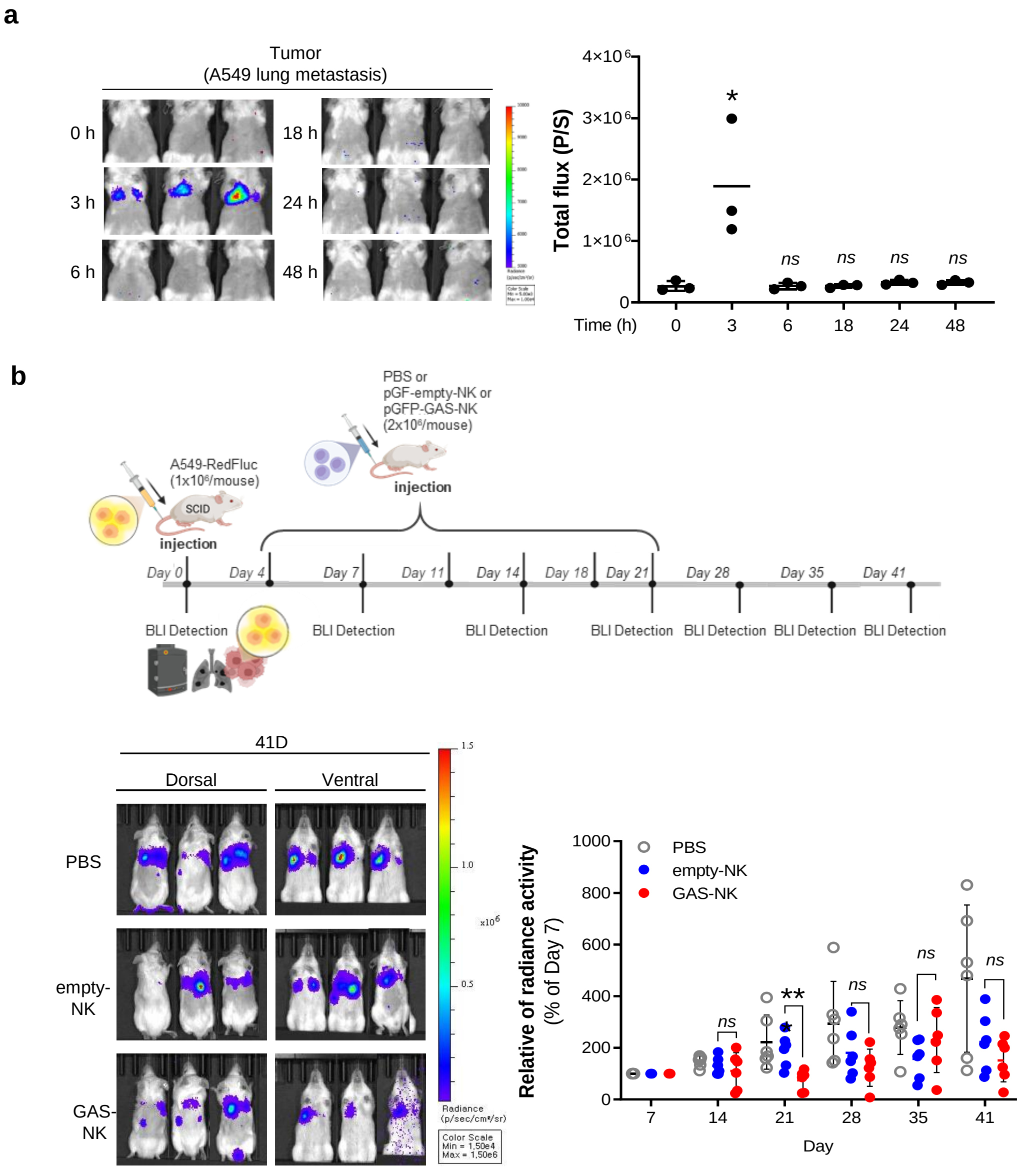

Supplementary Fig. 8. *In vivo* test for evaluating the anti-tumor effect of GFP-GAS-NK cells in an A549 lung metastasis model. (a) Time-lapse images depicting the BLI signal generated after intravenous injection of GAS-NK cells into A549 lung metastasis SCID mice. Quantitative data are presented alongside the images. Statistical significance is marked for observed differences in activation (*P < 0.05; ns, not significant). (n = 3, samples for all time points). (b) Schematic of the process of NK cell injection in the A549 lung metastasis tumor model for in vivo testing. Bioluminescence signals were calculated after background subtraction in total flux photons/sec from a body region of interest. Statistical significance is

indicated (*P < 0.05; ns, not significant). (n = 6, all other samples).

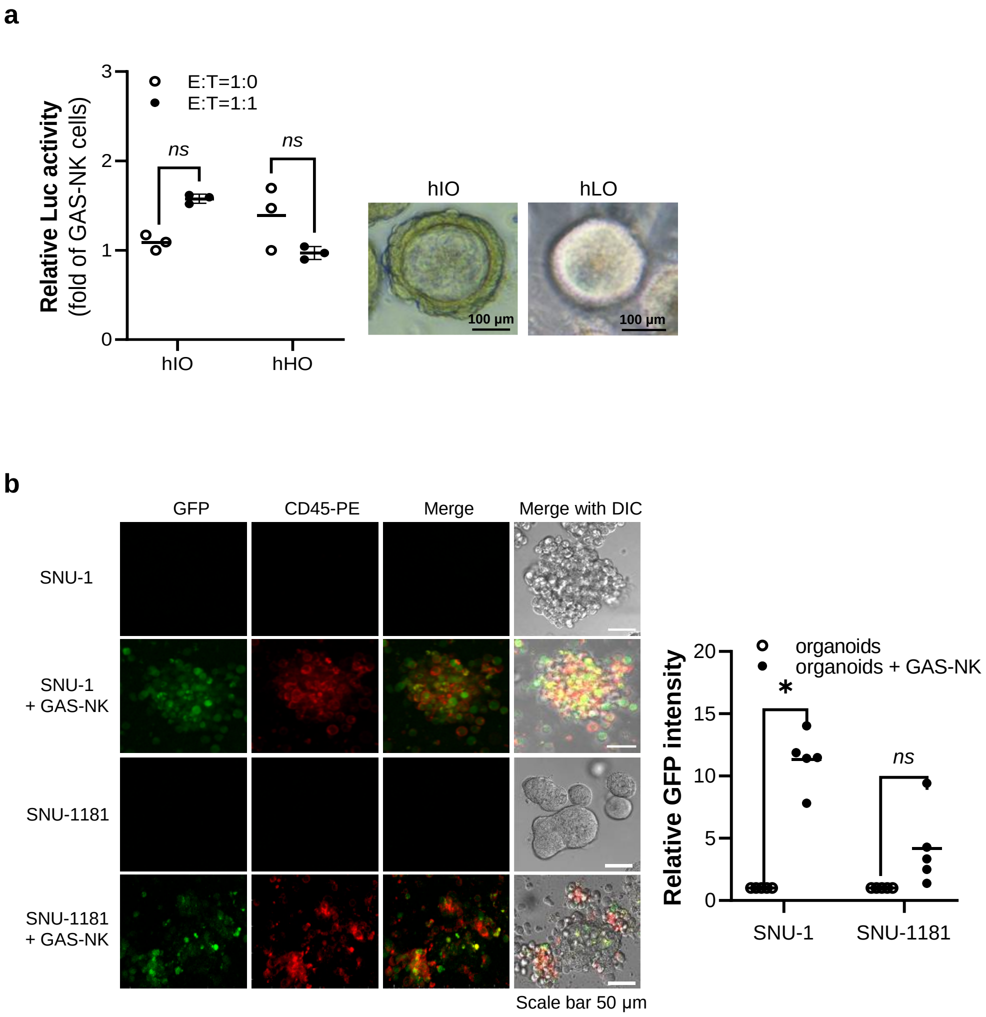

Supplementary Fig. 9. Activation efficacy analysis of GAS-NK cells in co-culture with human iPSC-derived organoids and patient-derived cancer organoids. (a) Graph of luciferase activity in GAS-NK cells after co-culture with human iPSC-derived liver and intestinal organoids, indicating no significant changes in activation (ns, not significant). (n = 3, all other samples). (b) Representative fluorescence images of GAS-NK cells (stained with GFP in green and CD45-PE in red) co-cultured with cancer organoids (SNU-1, SNU-1181) at an E:T ratio of 1:1, demonstrating GFP intensity. Statistical significance is marked for observed differences in activation (*P < 0.05). (n = 3, all other samples).

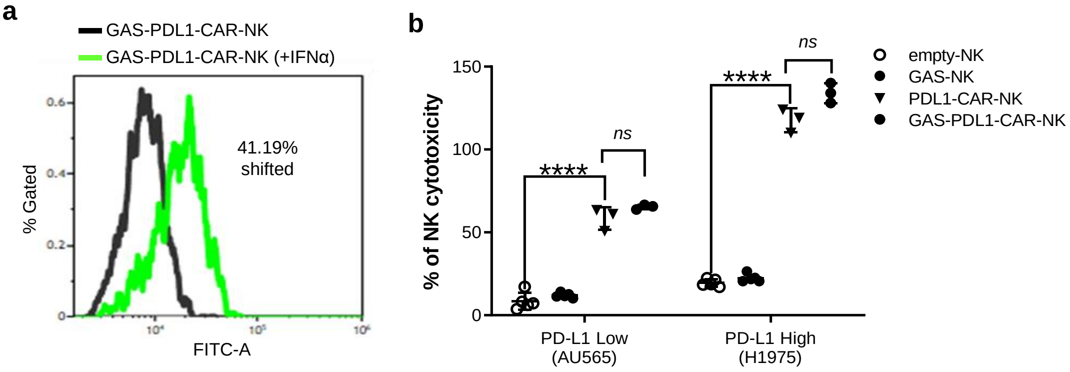

Supplementary Fig. 10. Application of GAS reporter system to primary NK and PDL1-CAR-NK cells. (a) Positive population of GFP-expressing GAS-PDL1-CAR-NK cells induced by IFNα. (n = 2, GAS-PDL1-CAR-NK and GAS-PDL1-CAR-NK treated with IFNα). (b) NK cell cytotoxicity to solid tumor cell lines. Cancer cell lines were treated with 5 µM of calcein AM for 30 minutes. After staining, cells were seeded into round-bottom 96-well plates at 5x10^4 cells per well. Empty-NK and GAS-NK cells were then serially increased in concentration and co-cultured for 4 hours. The cytotoxicity of the NK cell line was then determined. After co-culture, supernatants were recovered to measure calcein AM released from dead cancer cells using a fluorescence meter. Fluorescence was measured after dispensing cells into a 96-well black-sided, black-bottom plate, highlighting significant differences (****P < 0.0001; ns, not significant). (n = 3, all other samples).
